# The Role of Uniform Textures in Making Texture Elements Visible in the Visual Periphery

**DOI:** 10.1162/opmi_a_00136

**Published:** 2024-04-03

**Authors:** Marco Bertamini, Carolina Maria Oletto, Giulio Contemori

**Affiliations:** Department of General Psychology, University of Padova

**Keywords:** texture, crowding, peripheral vision, honeycomb illusion

## Abstract

There are important differences between central and peripheral vision. With respect to shape, contours retain phenomenal sharpness, although some contours disappear if they are near other contours. This leads to some uniform textures to appear non-uniform (Honeycomb illusion, Bertamini et al., 2016). Unlike other phenomena of shape perception in the periphery, this illusion is showing how continuity of the texture does not contribute to phenomenal continuity. We systematically varied the relationship between central and peripheral regions, and we collected subjective reports (how far can one see lines) as well as judgments of line orientation. We used extended textures created with a square grid and some additional lines that are invisible when they are located at the corners of the grid, or visible when they are separated from the grid (control condition). With respects to subjective reports, we compared the region of visibility for cases in which the texture was uniform (Exp 1a), or when in a central region the lines were different (Exp 1b). There were no differences, showing no role of objective uniformity on visibility. Next, in addition to the region of visibility we measured sensitivity using a forced-choice task (line tilted left or right) (Exp 2). The drop in sensitivity with eccentricity matched the size of the region in which lines were perceived in the illusion condition, but not in the control condition. When participants were offered a choice to report of the lines were present or absent (Exp 3) they confirmed that they did not see them in the illusion condition, but saw them in the control condition. We conclude that mechanisms that control perception of contours operate differently in the periphery, and override prior expectations, including that of uniformity. Conversely, when elements are detected in the periphery, we assign to them properties based on information from central vision, but these shapes cannot be identified correctly when the task requires such discrimination.

## INTRODUCTION

“A common man marvels at uncommon things. A wise man marvels at the commonplace.”

Confucius

Why do we see a grass meadow as green? One is tempted to respond in terms of photoceptors and ganglion cells in the retina responding to different wavelengths. But the L-M sensitivity (red-green channel) is extremely low at large eccentricities (Hansen et al., 2009). Our field of view is large and often filled with colourful scenes. Confucius may have been correct that a wise man can marvel at things that most people take for granted.

To understand that there is something to marvel at, it is useful to know more about the visual system. The fovea is the rod-free central region of the retina, covering less than 2 deg of the visual field. Outside the fovea rods greatly outnumber cones, colour sensitivity and acuity are therefore much reduced (acuity is about 50 times lower in the periphery, Anstis, 1998; Frisén & Glansholm, 1975). In general cone density is related to receptive field size and cortical magnification in V1 and V2, although there is greater magnification in the horizontal than the vertical direction and in the lower than the upper quadrant. Moreover, significant individual differences exist (Benson et al., 2021). Because information in the periphery is pooled over larger regions as a function of eccentricity (approximately linear) some differences in performance disappear with scaling (Pelli et al., 2004; Strasburger et al., 2011). In part, this addresses the question of colour vision for large uniform surfaces.

What is interesting is that we are hardly aware of any of these properties of the system from our subjective experience. The details and the colour of objects does not fade in the periphery of our field of view, and stimuli move to different locations retaining constant properties. It has been suggested that when we experience a detailed and uniform visual field this is a type of illusion. Because this illusion is part of our everyday experience, it has also been called the *grand* illusion (Blackmore et al., 1995; Noë et al., 2000; Rensink et al., 1997).

Recent research has rejected a simple view that peripheral vision is not useful at all in photopic vision. Instead, peripheral vision may be functionally different (Zhaoping, 2017) and more vulnerable to clutter (Rosenholtz, 2016). These issues are discussed in more detail later in the introduction. First, we briefly outline the aim of our study. We explored shape perception in the visual periphery using stimuli in which a grid masks the presence of small lines (Honeycomb illusion, Bertamini et al., 2016). The best-known situation in which context makes shapes harder to see in the periphery is crowding (Levi, 2008; Parkes et al., 2001). Here we are specifically interested in a related but different phenomenon. The main feature of the Honeycomb illusion is that the whole scene is uniform. Therefore, in addition to the masking effect of the context, there is the question of how the visual system deals with uniform textures, like the green meadow of our earlier example. Almost always, uniform textures are perceived as uniform. In the Honeycomb illusion this does not happen, and a uniform texture is perceived as non-uniform. We compared cases of uniformity and cases in which orientation of the lines changes from centre to periphery, and we used different tasks, so that in some cases observers were asked about their subjective experience, and in other they were asked a two alternative force-choice question. This way we can test for a dissociation between phenomenology (what people report seeing) and what people can identify.

### Central Vision Is for Shape Processing

As we have seen, there are important differences between central and peripheral vision in humans. It would be a mistake to characterize peripheral vision as simply a lower resolution version of central vision. For an extensive review of peripheral vision and shape perception see Strasburger et al. (2011), and for a review more specifically focused on the interaction between central and peripheral vision see Stewart et al. (2020).

Central and peripheral vision may have different functions. To use the terminology in Zhaoping (2019), peripheral vision is for looking and central vision for seeing. The idea is that detailed shape processing always involves foveal vision. This is consistent with the view that vision is active and eye movements are a key part of how we direct attention and see objects (Findlay & Gilchrist, 2003), and also with more recent evidence about foveal feedback. According to the foveal feedback hypothesis, objects that are presented away from fixation are nevertheless processed thanks to a feedback mechanism that carries that information to the foveal primary visual cortex (Chambers et al., 2013; Contemori et al., 2022; Fan et al., 2016; Williams et al., 2008).

Consistent with the idea that central vision is for processing shape and objects, observers are also more confident in their responses to central stimuli. This was shown using a task requiring observers to discriminate the orientation of gratings (Toscani et al., 2021). Conversely, when instead of perception of shape the task is about detection, observers are more confident in their responses to peripheral stimuli (Odegaard et al., 2018).

### Uniform Surfaces and Textures

We started with the example of a grass field. In this as in many other cases, an object or a texture extends from central to peripheral vision. This continuity is relevant because it provides useful information. When we look at the sky, or at the ocean, unless some boundary segments the surface, as in the case of a large white cloud, colour may fill the scene on the basis of absence of evidence against uniformity. For colour, filling-in phenomena can extend over large distances (Bressan et al., 1997; Pinna et al., 2003).

**Figure 1.**
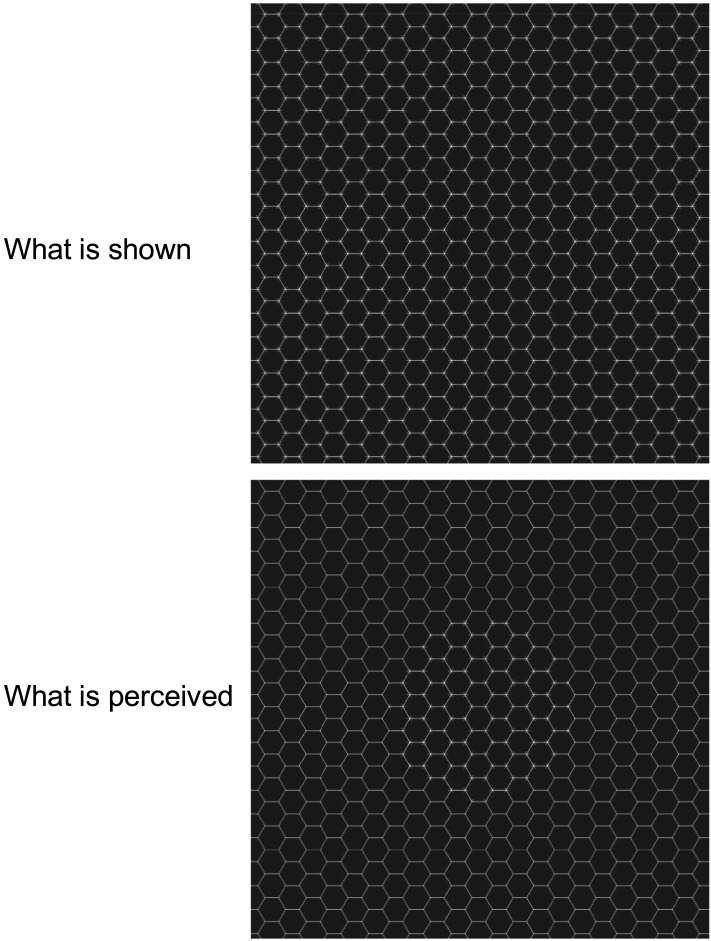
The texture on the left has hexagons and small lines at the vertices of the hexagons. While fixating the centre, observers see the lines in a region that does not extend to the whole texture. The image on the right was generated to illustrate the percept. This example may need to be enlarged, this and several other images are available for download: https://osf.io/kabyz/.

With respect to shape and objects, there are some special and counterintuitive phenomena, which have been called illusions. In the Honeycomb illusion (HI) a pattern with a regular structure, composed of a grid with additional features (small lines), the texture is perceived differently at fixation and in the periphery (Bertamini et al., 2016). Therefore, a physically uniform stimulus yields a non-uniform percept (see Figure 1). Because the experience does not change over time and with multiple fixations, intersaccadic memory seems to play no role.

Ninio and Stevens (2000) described a variation on the Hermann grid, in which disks present in the periphery completely disappear for the observers. They named this the Extinction illusion (EX). In this situation, just like in the HI, a regular pattern is perceived as different at fixation and in the periphery (see also Araragi & Kitaoka, 2011). Bertamini et al. (2019) have confirmed that the HI and EX are experienced by the majority of observers and are dependent on the location of the elements (i.e., the lines/disks have to be on the grid). They also found a dissociation between the two illusions in relation to contrast polarity, suggesting different mechanisms. In EX the disks have to be light grey on a dark background (e.g., black), or vice versa. In HI instead, the lines need to be the same colour as the grid.

Both illusions are instantaneous. Therefore, the disappearance of the disks and the lines is not an effect of adaptation, and is not related to fading phenomena such as Troxler effect (Martinez-Conde et al., 2004), or to the Uniformity illusion (Otten et al., 2017). Some experiments on a phenomenon closely related to the EX have been conducted by McAnany and Levine (2004) and by Levine et al. (2012). They called it “blanking” and found the minimum size of the grid necessary for the illusion (four squares) and confirmed that the luminance polarity of the disks and the grid has to be opposite.

### Summary Statistics

There is good evidence that in peripheral perception the visual system computes summary statistics (Chong & Treisman, 2005; Cohen et al., 2016; Haberman & Whitney, 2012; Lamme, 2010). This does not imply that all other information is lost. In the case of crowding, information about crowded stimuli is preserved at several stages of visual processing (Bornet et al., 2021; Herzog & Manassi, 2015; Manassi & Whitney, 2018). Summary statistics efficiently convey a wealth of information, like central tendencies, statistical dispersion, distribution shapes, and dependencies (Whitney & Yamanashi Leib, 2018). Summary measures might be computed in visual cortical areas by combining outputs from relevant pairs of V1-like cells and then applying averaging or pooling processes (Freeman & Simoncelli, 2011). This includes features like marginal luminance distribution, luminance autocorrelation, correlations of response magnitudes from oriented multi-scale wavelets (similar to those in primary visual cortex) across orientation, position, and scale differences, as well as phase correlations across scales (Portilla & Simoncelli, 2000).

Depending on the task, summary statistics can be very useful. It has been suggested that items that are unattended or are in the visual periphery are primarily perceived as being part of an ensemble (Cohen et al., 2016). Note, however, that in the case of the HI and EX, observers try their best to attend to the items away from fixation. Also, it is not clear to what extent observers are aware of summary statistics, and for this reason McClelland and Bayne (2016) have proposed a distinction between two types of summary statistics. Only the first type of summary statistics is information that observers are aware of.

A statistic representation that treats the image as a texture may provide a good approximation of the type of information encoded in the periphery, and it has been studied with paradigms such as crowding and visual search (Balas et al., 2009; Freeman & Simoncelli, 2011; Rosenholtz et al., 2012). In some cases, we can generate distortions in the periphery that observers do not notice, or multiple versions of images that appear the same. In this sense a non-uniform texture may appear uniform, or a distorted scene may appear normal and meaningful. The situation in the HI and EX is different. Here we are not interested in how well a texture captures a scene, but rather how a texture appears when it extends across the scene so that some of it is processed in central vision and some in peripheral vision. What is most relevant is to what extent what we perceive is what we expect, or what is most likely.

### Predictive Processing

This issue extends beyond extrapolation or interpolation of approximate peripheral information, as priors and predictive processing come into play (Friston, 2010; Rao & Ballard, 1999). A prior can originate in different ways. Regularities in the environment in which the system has evolved may be present as priors in the way the visual system works. Famous examples of this are the light-from-above assumption (Ramachandran, 1988) and the convexity assumption (Johnston et al., 1992). The same principle can apply to regularities encountered during learning, or from information carried over from one fixation to the next (transsaccadic memory, Blackmore et al., 1995; Deubel et al., 2002). Finally, some expectations may be linked to the present context. For example, from within an image a straight line may be more likely to continue straight than to change direction (an idea captured by the Gestalt principle of good continuation) (Wagemans et al., 2012).

Let us apply these priors to the case of the HI. Surprisingly, they all predict that the texture should be perceived as uniform. In terms of natural statistics, uniformity is a common property of textures. Even more surprising is the absence of any role of memory. One can see the texture before, and know that it is uniform. Importantly, multiple fixations across the texture provide ample evidence that at every fixation the lines are present. This should affect the system priors. Yet as soon as we move the eyes to a new location the lines that we just saw disappear. The readers are encouraged to test this for themselves, the images available on Open Science Framework can be seen on a large screen or printed on paper: https://osf.io/kabyz/.

How can we explain that we see the opposite of what we expect? The evidence shows that there are processes that govern perception that are not flexible, and do not change their behaviour with accumulated evidence. Some examples relate to amodal completion and were studied by Kanizsa (1980). The mechanism for perception of contours in the periphery differs from that in central vision, but still delivers sharp contours (Valsecchi et al., 2018). Indeed Galvin et al. (1999) found that a template for sharp edges is employed by the system when visibility is poor, and that this template is not affected by context.

### A Dissociation Between Subjective Report and Sensitivity

**Figure 2.**
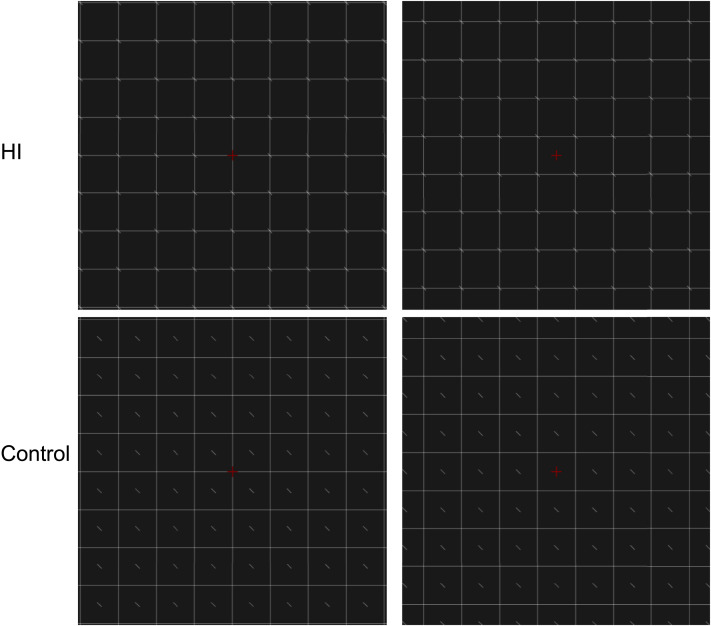
Illustration of the stimuli used in Exp 1a. In the HI condition the lines are at the corners of the squares, in the Control condition they are in the middle. There were also two positions of the grid (left and right columns), shifting the position of the lines and the relative position of the fixation cross. Note that distance from fixation in HI and Control is matched when one compares the diagonals.

**Figure 3.**
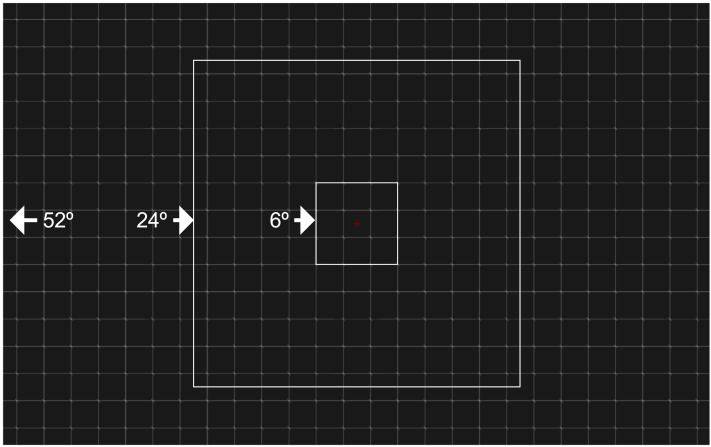
Participants had only three options, and were asked to choose the one that best captured what they perceived. Either the lines were visible over the whole screen (52° horizontally) or less than half of the screen (24°) and in a central region (6°). The lines were shown during the practice, but in the actual experiments the lines were not shown, and they relied on memory.

**Figure 4.**
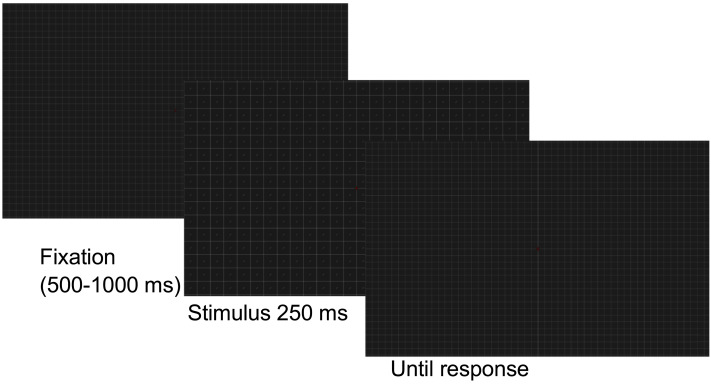
Illustration of the sequence of images in a trial. The empty grid used before and after the stimulus was a combination of the two positions of the grid (centered on fixation or not). Observers chose one of three possible responses (6, 12, 52 deg) to report how far from fixation they could see lines.

We report three experiments in which we manipulated whether the texture is objectively uniform (Exp 1a), or not (Exps 1b, 2, 3). We used a simplified HI with a square grid and lines at the corners of the squares, and we included a control condition with the same square grid and lines moved to the centre of the squares. The lines are equally likely to be tilted to the left or to the right. Examples of the stimuli are shown in Figure 2. Response options and Procedure are shown in Figures 3 and 4.

We predict a large difference in how far observers report seeing the lines in the HI and control conditions. This finding would replicate previous observations with new stimuli. In particular, it should replicate the results from Bertamini et al. (2019).

The presence of a central region in which the lines are oriented in a different way will allow a direct comparison between physically uniform and non-uniform textures (Exps 1a and 1b). We expect that the appearance of the lines in the visual periphery will not differ. This is because we think that the effect is due to how contours are perceived in the periphery, not because of extrapolation, or an interaction between centre and periphery. Once we will have established that we can use a non-uniform version of the HI, we will use non-informative lines in the central region (crosses, Exp 2) and ask people to try and judge whether the lines in the periphery are oriented to the left or to the right. For this task, unlike the previous one, there is a correct and incorrect answer, and we will be able to measure sensitivity.

As in the case of blindsight, observers may be able to detect some stimuli in a forced-choice task, even when they are not aware of their presence (Weiskrantz et al., 1974). Exp 2 will test whether some form of shape information is available to observers despite the illusory disappearance of the lines. Finally, Exp 3 will test whether the subjective disappearance can be explained by a bias in the report, by introducing a forced-choice task not about the shape identity but the presence or absence of the lines in the periphery. Table 1 summarises the hypotheses.

**Table 1.** Summary of the experiments including the predicted results.

Exp	Stimuli	Task	Prediction
Exp 1a	Uniform textures	How far are the lines visible in the periphery	Control: Over the whole area
	Lines at intersections (HI)		HI: in a central region only
	Lines in middle (Control)		

Exp 1b	Non-uniform textures	How far are the lines visible in the periphery	Control: Over the whole area
	Lines at intersections (HI)		HI: in a central region only
	Lines in middle (Control)		

Exp 2	Non-uniform textures	Lines oriented left or right (2AFC)	Fast decline with eccentricity in both HI and Control
	Lines at intersections (HI)		
	Lines in middle (Control)		

Exp 3	Pairs of stimuli, one uniform and one non-uniform	Uniform or non-uniform (2IFC)	Control: Good performance
			HI: Chance performance

## GENERAL METHODS

### Observers

Twelve volunteers participated in each of the three studies. All observers had normal or corrected-to-normal visual acuity. They were undergraduate or graduate students and were unaware of the purpose of the study. The study was approved by the local Ethics committee (Comitato etico della ricerca psicologica, Area 17, protocol 4855) and written consent was obtained from all participants.

### Stimuli and Apparatus

The images were generated using python and PsychoPy (Peirce, 2007) and displayed on a EIZO monitor (52.8 × 29.7 cm). The images filled the screen and had always a red cross in the centre. A chinrest was used to fix the distance from the screen at 57 cm.

The squares in the grid had always a side of 2 cm. All the lines were white (approximately 7.63 cd/m^2^) on a grey background (approximately 0.72 cd/m^2^). Figure 2 shows the key difference between HI and control condition, based on the location of the lines. It also shows a factor position of the grid necessary so that distance from fixation was matched for HI and Control conditions.

Before the study started there was a practice session with 32 trials. In the practice the lines specifying the response options were visible. Eye movements were monitored with an eyetracker (Gazepoint GP3), the stimulus was not presented if the participant did not look within a 2° radius from the fixation point. Presentation time was always 250 ms, and before and after the stimulus was shown a square grid remained visible. Viewing was binocular.

### Design

There were two illusion conditions (HI and Control), two orientations of the lines (left and right) and two positions of the grid (centered on fixation or not). There were 40 repetitions in Exp 1a, and therefore the total number of trials was 320. In Exp 1b and 2 there was an additional factor with 8 levels (size of inner region). The repetitions were only 5 and the total still 320.

## EXPERIMENT 1A

Twelve observers took part (age range: 19–55, mean age = 28.83, *SD* = 10.25, 9 females). In this study the texture was always uniform. The task was to report how far the lines were visible. There were 32 practice trails, identical to the experimental trials. Participants were not told any specific information about how many lines were present, and therefore did not know that lines were always present over the entire screen.

### Results

**Figure 5.**
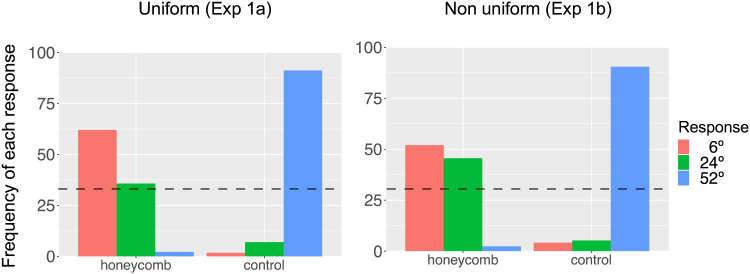
Observers chose one of three sizes to report how far from fixation they could see lines. There were only 3 possible responses (6, 12, 52 deg), shown with different colours in the graph. Here we plot the frequency (in %) of the responses in the two conditions (HI, Control). Chance level (33%) is shown as a dashed line. Left and right panels show data from Experiment 1a and 1b.

**Figure 6.**
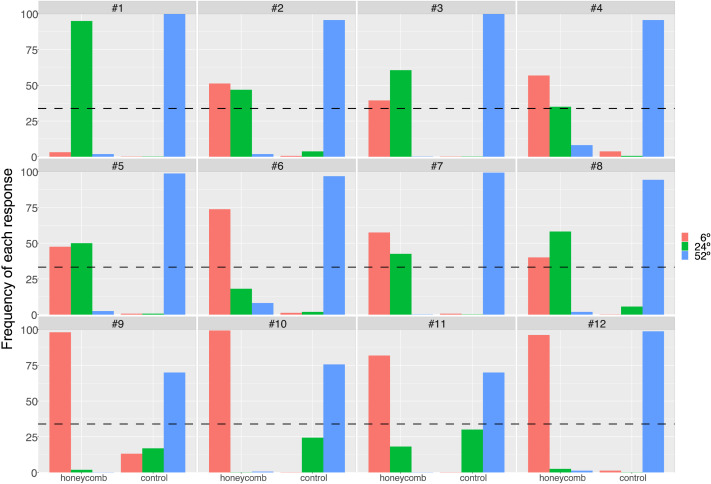
Observers chose one of three sizes to report how far from fixation they could see lines. There were only 3 possible responses (6, 12, 52 deg). Here we plot the frequency (in %) of the responses in the two conditions (HI, Control). Chance level (33% for each of the three options) is shown as a dashed line. Panels show individual data.

Percentage of responses are shown in Figure 5. Individual data is shown in Figure 6. In the Control condition the most common response was that the lines were visible over the entire screen (52°). By contrast, in the HI condition the most common answer was that the lines were only visible in the central region (6°). Since the extent of the region with visible lines could only be estimated from a limited number of options, we used a Poisson generalised mixed effect model, with the count of each of the three possible responses as the dependent variable and Condition (HI, Control) as factor. We confirmed the difference between the two conditions (*χ*^2^ = 28.7, *p* < 0.001). Further tests will be reported together with Exp 1b. We verified model assumptions using the DHARMa package in R (Diagnostics for Hierarchical Regression Models). The dispersion test yielded a significant result (*p* < 0.001), suggesting that the observed data exhibited lower dispersion than anticipated based on the fitted model.

We think the model does capture the important differences, and we checked here and in all our experiments, whether the same factors are significant in a non-parametric analysis (Aligned Ranks Transformation ANOVA, ART) (Wobbrock et al., 2011). This ANOVA confirmed the difference between the two conditions (*F*(1, 11) = 173.59, *p* < 0.001, *η*^2^ = 0.94).

## EXPERIMENT 1B

Twelve observers took part (age range: 20–26, mean age = 22.58, *SD* = 1.93, 9 females). In this study the texture was never uniform, because the lines in the central region were oriented in the opposite way with respect to the lines in the outside of this central region. In every other respect the study was the same as Exp 1a. This manipulation will allow us to directly compare uniform and non-uniform textures.

**Figure 7.**
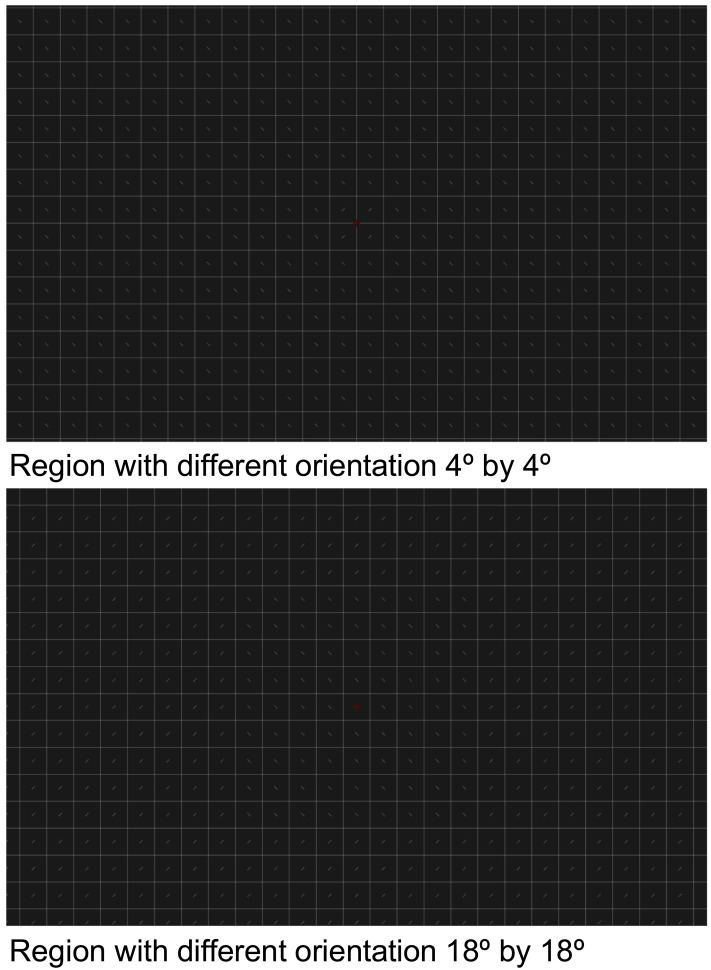
The region in which the lines had a different orientation could extend from a minimum width of 4° to a maximum of 18°. Here we show just these two cases for the Control condition.

There were 8 values for the side of the central square region: 4, 6, 8, 10, 12, 14, 16, and 18°. The smallest and the largest regions are shown in Figure 7. These values are relative to the midpoint between features with one orientation and features with another orientation. Because the question for the observers was to report how far they could see lines, the change in orientation was irrelevant for the task.

### Results

Percentage of responses are shown in Figure 5. In the Control condition the most common response was that the lines were visible over the entire screen (52°). By contrast, in the HI condition the most common answer was that the lines were only visible in the central region (6°).

**Figure 8.**
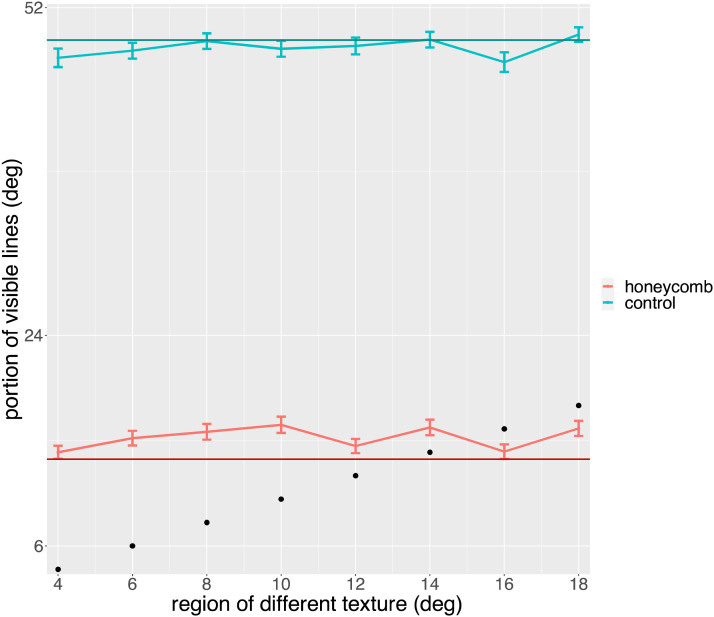
Observers reported how far from fixation they could see lines. They chose one of three possible responses (6, 12, 52 deg) and here we plot the mean of these responses. The graph shows the means in the two conditions (HI, Control) as a function of the size of the region with different texture. The size of this region is also marked by black dots. The solid horizontal lines show means from Exp 1a.

In Figure 8 we plot the mean response as a function of the size of the region with different texture. It is clear that this irrelevant dimension did not affect the responses. It is also clear that the mean is very close to that of Exp 1a (superimposed as a solid line). We tested for an effect of Size of region treated as continuous, with a Poisson linear mixed effect model in which we included Condition (HI, Control). We confirmed the effect of condition (*χ*^2^ = 20.86, *p* < 0.001), but there was no effect of the Size of the region (*χ*^2^ = 0, *p* = 0.95) or interaction (*χ*^2^ = 0.58, *p* = 0.44). The DHARMa nonparametric dispersion test was significant (*p* < 0.001). A non-parametric analysis (Aligned Ranks Transformation ANOVA, ART) confirmed that the only significant difference was that between the two conditions (*F*(1, 165) = 977.09, *p* < 0.001, *η*^2^ = 0.86). Note that in this analysis the Size of the region was ordered rather than continuous.

To compare 1a and 1b we performed a mixed ANOVA with the following factors: Condition (HI, Control), Line orientation (Left, Right), Grid position (On fixation, Off fixation), and finally the Uniformity of the texture as a between-subjects factor (Exp 1a: Uniform, Exp 1b: Non-uniform). The size of the region with lines in opposite direction was not included as it was only a factor in Exp 1b. We confirmed an effect of condition (*χ*^2^ = 99.88, *p* < 0.001) and no other effects or interactions. Again, the non-parametric analysis (Aligned Ranks Transformation ANOVA, ART) confirmed that the only significant difference was that between the two conditions (*F*(1, 161) = 1290.88, *p* < 0.001, *η*^2^ = 0.89).

In summary, Exps 1a and 1b confirm the basic phenomenon, the disappearance of lines that makes a uniform texture appear non uniform. In addition, the comparison of the two studies shows that there is no major role for the information in the central region. In other words, the physical uniformity (Exp 1a) is not important and does not make the texture appear more uniform.

## EXPERIMENT 2

Twelve observers took part (age range: 18–56, mean age = 25.50, *SD* = 10.09, 9 females). In this study the texture was not uniform. We used crosses (both lines superimposed) in the central region, because we wanted to make sure this region had no information about orientation. The lines outside the central region, as before, were either tilted left or tilted right with equal probability.

Experiment 2 had two parts. The first part had the same procedure as Exp 1a, participants had to report how far they could see lines. The second part was different. Here participants were told that in the outside the lines were tilted either left or right, and they had to respond with a forced-choice between left and right orientation. If unsure they were told to guess. Participants were not told that the stimuli in the two parts of the study were identical, and the only difference was the randomization of the trials and the task.

### Results

**Figure 9.**
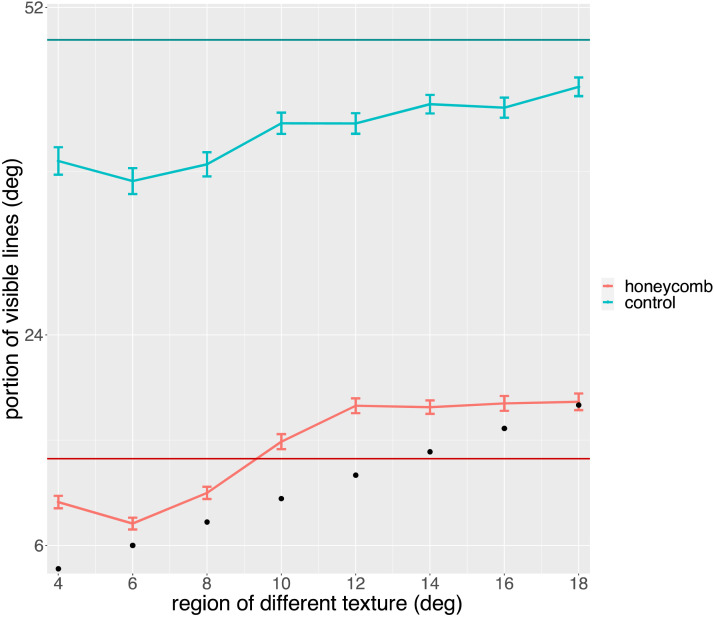
Observers reported how far from fixation they could see lines. They chose one of three possible responses (6, 12, 52 deg) and here we plot the mean of these responses. Data from the first part of Exp 2. The graph shows the means in the two conditions (HI, Control) as a function of the size of the region with different texture. The size of this region is also marked by black dots. The solid horizontal lines show means from Exp 1a.

In Figure 9 we plot the mean response as a function of the size of the region with different texture. The effect of condition is similar to that of Exp 1a and 1b, with a much larger extent for the HI. As in Figure 8, the mean for Exp 1a is superimposed. However, there now seems to be an effect of the region with a different texture. We tested for an effect of size of region with a Poisson generalised linear mixed model in which we included Condition (HI, Control). The DHARMa nonparametric dispersion test was non-significant (*p* = 0.100). We confirmed the effect of condition (*χ*^2^ = 8.19, *p* = 0.004), an effect of the size of the region (*χ*^2^ = 4.97, *p* = 0.026) and their interaction (*χ*^2^ = 4.67, *p* = 0.031). As can be seen in Figure 9, more lines were seen as the number of crosses increases.

A non-parametric analysis (Aligned Ranks Transformation ANOVA, ART) confirmed the significant effect of Condition (*F*(1, 165) = 690.58, *p* < 0.001, *η*^2^ = 0.81) and of size of the region (*F*(1, 165) = 6.48, *p* < 0.001, *η*^2^ = 0.22), but found no significant interaction. As before, we note that in this case the size of the region was ordered rather than continuous.

The reason participants said they could see more lines when there were more crosses can be explained by the fact that more crosses does mean more lines overall. Moreover, crosses are twice as large (two lines) and brighter than single lines. This creates a central region in which the lines are salient, and as the region increases so do the average response about how far lines are visible. In other words, the crosses provide a pedestal. This effect of region therefore does not change substantially the conclusions from Exp 1a and Exp 1b.

**Figure 10.**
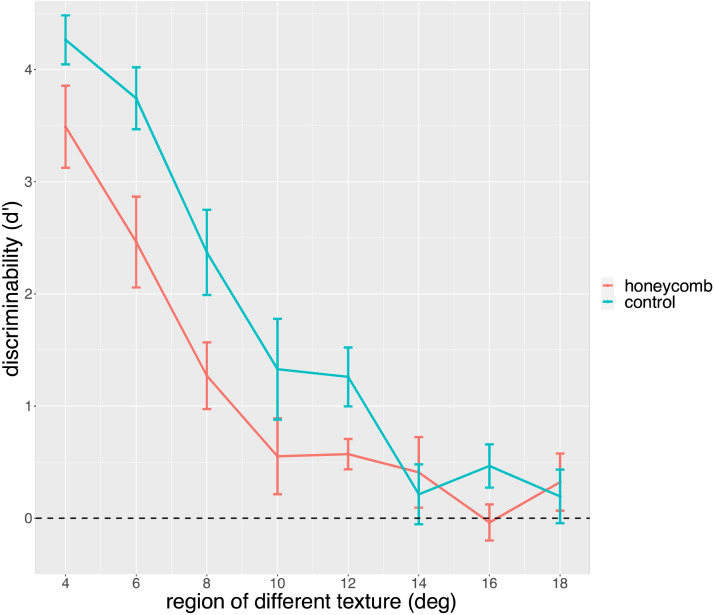
Mean *d*′ scores in the two conditions (HI, Control) as a function of the size of the region with different texture. Observers reported the orientation of the lines (2AFC). Data from the second part of Exp 2.

We now turn to the analysis of the second part of Exp 2. We can use the response given to compute an index of sensitivity, on the basis of signal detection theory. The *d*′ is a bias free measure of sensitivity to a signal. Mean *d*′ values are shown in Figure 10. When the central region is small, people can see the lines in the outside region and their performance is extremely good. As the central region gets larger *d*′ drops fast, and is not different from zero already around 14°. That is to say, observers are not able to discriminate above chance line orientations if these lines are farther than 7° from fixation. Importantly, this point in the graph is the same for the HI and the Control condition.

We analysed mean *d*′ values with a linear model in which we included the size of region and Condition (HI, Control). We confirmed the effect of condition (*F*(1, 177) = 221.96, *p* < 0.001, *η*^2^ = 0.56), an effect of the size of the region (*F*(1, 177) = 13.11, *p* < 0.001, *η*^2^ = 0.07) and their interaction (*F*(1, 177) = 5.67, *p* < 0.001, *η*^2^ = 0.03).

A non-parametric analysis (Aligned Ranks Transformation ANOVA, ART) confirmed the significant effect of Condition (*F*(1, 165) = 22.52, *p* < 0.001, *η*^2^ = 0.12), and effect of size of the region (*F*(1, 165) = 35.75, *p* < 0.001, *η*^2^ = 0.60), and their interaction (*F*(1, 165) = 2.89, *p* = 0.007, *η*^2^ = 0.11).

At this point it is interesting to compare the results from the two parts of Exp 2. Observers were asked whether they saw lines, not about how well they could see them or whether they could see the shape as well in the centre and in the periphery. On the other hand, if a texture appears uniform this implies, logically, that the lines are the same at different locations. The comparison between Exp 1a and 1b already suggests that the ability or inability to see lines in the periphery is not directly related to how uniform the texture appears, because changing the appearance in the central region had no effect on the responses. What about the relationship between seeing lines and seeing their orientation. Based on the large difference between HI and Control, replicated in all three experiments, one might expect better performance when the lines are in the middle of the square. On the other hand, in the control condition the phenomenal sense of existence of lines over the whole pattern may have been illusory, in the sense that observers were unaware of the difference between the lines in the central region (with a definite orientation) and lines in the periphery (present but not in a way that their shape could be identified).

Given that *d*′ prime drops sharply and performance is very poor beyond 14 degrees, this suggests that in the HI what people see and what they can identify matches fairly well, but for the Control condition there is a dissociation, lines are reported over a region in which identification is not possible. This difference in detectability does not seem to destroy the impression of a uniform texture.

## EXPERIMENT 3

Experiment 1a and 1b confirmed that observers do not see lines in the periphery when the lines are at the intersection of a grid. The task was to report how far the lines were visible. This kind of procedure is designed to capture the phenomenal appearance, and has all the limitations of a task in which there is no right or wrong answer. Participants may be biased to say that they do not see lines for various reasons. A different way to access what people see is to force them to choose which image has a uniform texture. If the lines are not visible in the periphery, it would be very difficult to select the image in which lines are missing. Experiment 3 was designed exactly to collect these data.

We used the same images as those of Experiment 1a (Honeycomb and Control conditions) but with the following changes. We created pairs of images, one was uniform (lines present over the entire image) and one was not uniform (lines removed starting from an eccentricity of 22 degrees). This distance may seem arbitrary. However, the important comparison, as before, is between the HI and the Control condition. We predict a large performance difference.

Note that presence or absence of lines changes overall brightness. There are therefore possible strategies that observers could use. We introduced variability in contrast by changing the brightness of the lines. The low contrast condition had lines of 3.91 cd/m^2^, the high contrast was the same as the previous experiments. We reasoned that performance may be higher for higher contrast if observers used a strategy based on brightness.

During the inter-stimulus period there was a dynamic grid (the same grid as in the stimulus, but translated twice). This dynamic grid was presented for 100 ms. In each critical interval the stimuli were presented for 150 ms. Design was 2 (contrast high vs low) × 2 (interval of non-uniformity first vs second) × 2 (condition honeycomb vs control) × 2 (grid position) × 2 (lines orientation left vs right). Every trial was repeated 10 times for a total of 320 trials.

Twelve naive observers took part (age range: 18–37, mean age = 22.50, *SD* = 4.89, 11 females). Participants were asked to report in which of the two intervals the grid was uniform.

### Results

**Figure 11.**
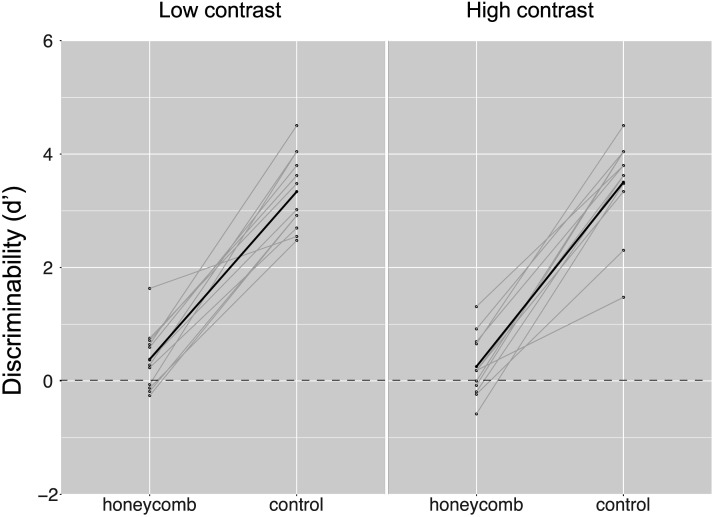
Observers reported the uniformity or non-uniformity of the whole stimulus (2ICF). Sensitivity (*d*′) is shown in the two conditions (HI, Control) and for two contrasts (low vs high). Grey lines represent data from single participants, and the black line is the mean.

In Figure 11 we plot the *d*′ separately for the low and high contrast and for the Honeycomb and the control condition.

We conducted a linear mixed model type III ANOVA with condition and contrast as fixed factors. The results showed an effect of condition (*F*(1, 33) = 404.055, *p* < 0.001), no effect of contrast (*F*(1, 33) = 0.015, *p* = 0.902) and no interactions (*F*(1, 33) = 0.917, *p* = 0.345). The *d*′ for the HI condition was 0.32, whereas for the Control condition was 3.42. These results suggest that participants could not reliably discriminate between a uniform and a non-uniform texture in the HI condition, but they could do it for the control condition.

There was no evidence of a strategy based on brightness, because there was no effect of contrast. The important implication of these results is that the difference found in previous studies, and in particular in Exp 1a and 1b was not a bias in the subjective report. That is, even if observers were to expect, for whatever reason, to see fewer lines in the HI case, they might have been able to report these lines when they were forced to choose between two alternatives (presence or absence of the lines).

## DISCUSSION

In the honeycomb illusion (HI) textures that are uniform appear non-uniform. Something similar happens in the extinction illusion (EX), where elements disappear away from fixation (Ninio & Stevens, 2000), and in the redundancy masking effect (Yildirim et al., 2020), where number of elements is underestimated. These are all strong effects. We believe the Honeycomb illusion is particularly interesting because observers see an illusory change in appearance of an extended surface/texture. The effect is therefore not based on context (features are the same at every location), not based on a prior for uniformity, and not based on memory (Bertamini et al., 2016).

In three experiments using the HI we confirmed that when lines are presented on top of a grid, they are visible in a central region, and they disappear in the periphery. For the version that we used, with a square grid, the central region in which participants reported seeing lines was approximately 14 degrees of visual angle (an eccentricity of 7 degree from fixation). The procedure was not designed to obtain a precise measure, and the size of the region is likely to vary with several parameters, for example the length of the lines (Bertamini et al., 2016). Importantly, the lines remained visible over the entire screen (52 degrees in width) in the control condition. The only difference between illusion and control condition was the location of the lines with respect to the grid as in the control condition, they were positioned in the middle rather than at the corners. The small size of the lines is therefore not in itself an explanation for their disappearance.

One new observation in this study was a direct comparison between a uniform and a non-uniform texture. In Exp 1a the texture had lines leaning to the left in some trials or to the right in other trials. In Exp 1b the texture had an inner region in which the lines were oriented differently from the outer region. This manipulation had no effect on the visibility of the lines reported by the observers. Regularity is therefore not a critical aspect of the Honeycomb illusion, supporting the hypothesis that what is perceived in the periphery is largely a result of how shape is processed in the periphery independently from what is perceived at fixation.

So far, we have studied and commented on what we called the region of visibility. This refers to a subjective report about phenomenal presence of the lines. In Exp 2 we had two blocks, the first was the same as in Experiment 1, the second instead asked participants to decide whether the lines were tilted to the right or to the left (forced-choice task). Although this was hard, we encouraged observers to try and, if necessary, make a guess. The aim was to test the presence of sensitivity to shape beyond phenomenal awareness. Subjective reports and discrimination performance may dissociate, as in the case of blindsight (Weiskrantz et al., 1974).

Performance, as indexed by *d*′ scores, was very high as long as the lines were not too far from fixation, and only slightly higher for the control condition. However, performance dropped sharply as soon as the lines were more than 14 degrees away from fixation. After this distance, there was no difference between HI and control condition, despite the fact that in the control condition the lines were placed in the middle of the square.

Results show that in the illusion condition the size of the region in which lines are seen and the size of the region in which they can be identified are in good agreement. Although again we have to stress that the procedure was not one that allowed precise estimation. In the control condition instead, there was a large dissociation between subjective and objective measures. We suggest that this is further evidence of how contours in the periphery are processed differently from central vision, but that phenomenally they appear clear and well defined (Galvin et al., 1999; Valsecchi et al., 2018). In a sense, the dissociation in the control condition could be described also as an illusion, related to the general "grand illusion" of peripheral vision (Blackmore et al., 1995; Dennett, 1991). The fact that in the control condition lines are seen, but their orientation cannot be reported is similar to what happens in the crowding phenomenon. In crowding is the identification rather than the presence of an object that is impaired (Levi, 2008; Parkes et al., 2001; Pelli & Tillman, 2008). However, more work is necessary to explore how confident observers are in their perception of oriented lines. If they are, but they are unable to judge their orientation, this could be described as a case of blindsense (Garric et al., 2019).

Experiment 3 was a control for the findings of Experiment 1. We expected that even when offered a forced choice between presence and absence of the lines, observers could only rely on their subjective perception. This was confirmed because observers could not discriminate between stimuli in the HI case. They had no problem instead in reporting the presence of the lines the control condition.

Overall, the results from our study demonstrate that extrapolation is not a general solution to peripheral vision limitations. In the case of textures, extrapolation would seem even more likely than for complex scenes, and yet in the honeycomb illusion extrapolation fails. Note that the control condition shows a sense of presence of lines unrelated to identification. Therefore, one could expect that also for the regular texture presented in the honeycomb illusion, the system might extrapolate the presence of lines, as the problem of identification is not a necessary factor. Instead, no extrapolation takes place, either immediately or over time. What contours we perceive is rigidly determined by how contours are computed in the peripheral region of the visual field.

## AUTHOR CONTRIBUTIONS

M. B. and C. M. O., Conceptualization. M. B. Writing – original draft preparation. M. B., C. M. O., and G. C., Writing – review and editing. M. B., C. M. O., and G. C., Analyses. All authors have read and agreed to the published version of the manuscript.

## FUNDING INFORMATION

The research was in part supported by a Grant (Ricerca scientifica di eccellenza) from CARIPARO Fundation on “Recognising shapes: a full investigation of a novel entrainment procedure”.

## DATA AVAILABILITY STATEMENT

Data was collected between October 2022 and August 2023. All datafiles are made available on OSF: https://osf.io/g9d6c/.
